# Radiomics in Oncology III

**DOI:** 10.3390/diagnostics13010149

**Published:** 2023-01-01

**Authors:** Marta Zerunian, Andrea Laghi, Damiano Caruso

**Affiliations:** Radiology Unit, Department of Medical-Surgical Sciences and Translational Medicine, Sant’Andrea University Hospital, “Sapienza” University of Rome, 00189 Rome, Italy

In recent years, radiomics has been among the most impactful topics in the research field of quantitative imaging. Radiomics is a method applied to imaging that enables the extraction of thousands of quantitative ultrastructural parameters from pixels, which are neither assessable nor quantifiable by the human eye. This approach has allowed access to a large amount of data on tissue heterogeneity and neighboring pixel behavior, with potential impacts on lesion characterization in terms of aggressiveness, prognostic value and response to treatment. Another important aspect compared to the other “omics” branches is the capability to extract such information directly from routine examinations without the need for further analysis, as is needed for other “omics” such as proteomics or genomics. This fundamental strength of radiomics has led to a huge impact on research in the radiology field with almost 6000 papers published since 2012.

Oncology is one of the most explored fields with radiomics, with interesting results from diagnosis to treatment and follow-up in many different tumor types and anatomical districts [1,2]. However, one of the emerging criticisms of radiomics publications is the lack of standardization and the need for more data and validation studies. In fact, it is important to support the knowledge acquired with large cohorts and innovative perspectives in order to improve the standardization process.

For instance, in the previous Special Issue “Radiomics in Oncology II”, several oncologic topics were developed and explored.

In the field of lung cancer, an interesting perspective is provided by Thattaamuriyil Padmakumari L and colleagues on the capability of radiomics to differentiate lung cancer from tuberculosis detected on a chest CT scan; the potential of the research is very promising, enabling characterization of the lesion in a rapid and non-invasive manner, which is particularly helpful in many poor nations, improving the management of oncology patients [3].

Another important step in the management of lung cancer and radiomics is present in this Special Issue with the research of Rundo L and colleagues; they investigated the potential impact of radiomics on the characterization and risk stratification of the indeterminate prevalent pulmonary nodule assessed with low-dose CT for lung cancer screening. Also with the help of machine learning, the study showed how radiomics might improve the characterization of pulmonary nodules in the near future, with concrete implications for clinical practice in terms of screening recall interval optimization and targeted management of patients [4].

With the aim of advancing to clinical use faster, Castaldi R and colleagues tested an ad hoc weighted statistical framework to investigate the effect of different normalization methods of the principal component and downstream analysis in order to generate a reliable radiomic signature to characterize the different breast cancer phenotypes extracted from both MRI and PET imaging [5].

Shifting to response to treatment, Cheng S and colleagues propose an interesting paper regarding the capability of radiomics to differentiate response to therapy and time to progression in metastatic liver lesions from pancreatic malignancy. Their results show how some radiomic parameters correlate with survival data better than conventional imaging evaluation criteria such as RECIST 1.1, Choi or modified Choi [6]. Then, a detailed radio-pathological case report of a pancreatic colloid carcinoma from Chen C H and colleagues is presented, which is helpful to deepen understanding of the structure of a neoplastic lesion in order to correctly interpret its transposition into imaging and radiomics [7].

We hope that readers appreciated the previous Special Issue and will appreciate the content of this new Special Issue “Radiomics in Oncology III” even more, which elucidates further steps in imaging data extraction and future applications of radiomics in the clinical setting, which remains crucially important for patient management in the era of personalized medicine.
